# Supplementary material for: Methodology and applicability of the human contact burn injury model: A systematic review
Source: PLoS One. 2021 Jul 30;16(7):e0254790. doi: 10.1371/journal.pone.0254790 (PMC8323928; doi:10.1371/journal.pone.0254790)
Supplement: S1 Table — (DOCX) [file pone.0254790.s002.docx]

**S1 Table. Statistical definitions.**

| Agreement [1] | Agreement between two methods or raters of clinical measurement is quantified using the differences between observations made using the two methods on the same subjects. |
| --- | --- |
| Coefficient of variation [2] | Standard deviation of the differences between pairs of measurements divided by the mean of measurements in the compared samples. |
| Reliability [3-5] | The measurement can be obtained with stated precision by a different team using the same measurement procedure, the same measuring system, under the same operating conditions, in the same or a different location on multiple trials.  Formally defined as:  $\frac{{(SD of subject^{'}s true values)}^{2}}{{(SD subjects^{'}true values)}^{2}+{(SD measurement error)}^{2}}$  also known as an intraclass correlation coefficient (ICC) defined as the proportion of the total variance of an observation that is associated with the class (e.g., subject) to which it belongs. |
| Repeatability [3,6] | The measurement can be obtained with stated precision by the same team using the same measurement procedure, the same measuring system, under the same operating conditions, in the same location on multiple trials.  Repeatability for pairs of repeated observations during the same conditions is two times the standard deviation of the differences between them; two determinations will differ by less than the repeatability with 95% confidence. |
| Reproducibility [3,4] | The measurement can be obtained with stated precision by a different team, a different measuring system, in a different location on multiple trials. Thus, reproducibility is the degree of the variation in measurements made on a subject under changing conditions. |
| Variability [5] | Numerical difference between test-retest data divided by the average of both sessions expressed as a percentage. |

1. Bland JM, Altman DG. Measuring agreement in method comparison studies. Stat Methods Med Res. 1999;8(2):135-60. doi: 10.1177/096228029900800204.

2. Pedersen JL, Kehlet H. Hyperalgesia in a human model of acute inflammatory pain: A methodological study. Pain. 1998;74(2-3):139-51. doi: 10.1016/s0304-3959(97)00160-7.

3. McArthur SL. Repeatability, Reproducibility, and Replicability: Tackling the 3R challenge in biointerface science and engineering. Biointerphases. 2019;14(2):020201. doi: 10.1116/1.5093621.

4. Bartlett JW, Frost C. Reliability, repeatability and reproducibility: analysis of measurement errors in continuous variables. Ultrasound Obstet Gynecol. 2008;31(4):466-75. doi: 10.1002/uog.5256.

5. Varrone A, Fujita M, Verhoeff NP, Zoghbi SS, Baldwin RM, Rajeevan N, et al. Test-retest reproducibility of extrastriatal dopamine D2 receptor imaging with [123I]epidepride SPECT in humans. J Nucl Med. 2000;41(8):1343-51.

6. Bland JM, Altman DG. Statistical methods for assessing agreement between two methods of clinical measurement. Lancet. 1986;1(8476):307-10.
