# Supplementary material for: Methodology and applicability of the human contact burn injury model: A systematic review
Source: PLoS One. 2021 Jul 30;16(7):e0254790. doi: 10.1371/journal.pone.0254790 (PMC8323928; doi:10.1371/journal.pone.0254790)
Supplement: S1 File — (DOCX) [file pone.0254790.s003.docx]

# **S1 File. Embase search terms.**

1. exp hyperalgesia/
2. exp pain threshold/
3. exp perceptive threshold/
4. sensitization/
5. exp pain measurement/
6. exp temporal summation/
7. 1 or 2 or 3 or 4 or 5 or 6
8. (hyperalgesia or pain threshold or detection threshold or pain sensitization or pain measurement or temporal summation).mp. [mp=title, abstract, heading word, drug trade name, original title, device manufacturer, drug manufacturer, device trade name, keyword, floating subheading word, candidate term word]
9. 7 or 8
10. exp thermal stimulation/
11. exp heat injury/
12. exp thermal injury/ or burn injury.mp.
13. thermode.mp.
14. 10 or 11 or 12 or 13
15. (contact heat stimulation or heat injury or burn injury or local hyperthermia or thermode).mp. [mp=title, abstract, heading word, drug trade name, original title, device manufacturer, drug manufacturer, device trade name, keyword, floating subheading word, candidate term word]
16. 14 or 15
17. normal human/
18. exp volunteer/
19. human/
20. patient/
21. 17 or 18 or 19 or 20
22. (Healthy subjects or healthy volunteers or human subjects or humans or man or patients).mp. [mp=title, abstract, heading word, drug trade name, original title, device manufacturer, drug manufacturer, device trade name, keyword, floating subheading word, candidate term word]
23. 21 or 22
24. 9 and 16 and 23
