# Supplementary material for: Methodology and applicability of the human contact burn injury model: A systematic review
Source: PLoS One. 2021 Jul 30;16(7):e0254790. doi: 10.1371/journal.pone.0254790 (PMC8323928; doi:10.1371/journal.pone.0254790)
Supplement: S2 File — (DOCX) [file pone.0254790.s004.docx]

**S2 File. Results from the “miscellaneous” studies.**

#### **Study characteristics**

Two studies examined the analgesic effects of placebo on heat pain [1,2]. A total of 92 subjects with a gender ratio (male/female) of 1.1 (48/44) was included. None of the studies reported anthropometrics nor sample size estimates.

#### **Study outcomes**

In one randomized crossover CBI study, subjects received either inactive placebo (subjects were told the placebo was a strong analgesic) or received no treatment at all [1]. The results showed reduced pain ratings during heating in the placebo condition, as well as reduced heart-rate variability and subjective stress. Another study investigated whether a sham magnet treatment, told to alleviate pain, could reduce SHA after a CBI [2]. The results suggested less pronounced SHAs with the sham magnet, as well as reduced pain ratings during the CBI.

1. Aslaksen PM, Flaten MA. The roles of physiological and subjective stress in the effectiveness of a placebo on experimentally induced pain. Psychosom Med. 2008;70(7):811-8. doi: 10.1097/PSY.001361818105ed.

2. Matre D, Casey KL, Knardahl S. Placebo-induced changes in spinal cord pain processing. J Neurosci. 2006;26(2):559-63. doi: 10.1523/jneurosci.4218-05.2006.
