# Supplementary material for: Methodology and applicability of the human contact burn injury model: A systematic review
Source: PLoS One. 2021 Jul 30;16(7):e0254790. doi: 10.1371/journal.pone.0254790 (PMC8323928; doi:10.1371/journal.pone.0254790)
Supplement: S3 File — (DOCX) [file pone.0254790.s005.docx]

**S3 File. The contact burn injury model in comparison to other cutaneous heat pain models.**

**Study outcomes from the methodological studies**

One study compared temporal summation to different stimulus modalities between the CBI-model and the intradermal capsaicin model. Both models induced temporal summation in the SHA with few differences, but these changes were more pronounced in the capsaicin model [1]. The effect of thermal pre- and post-CBI conditioning was compared between the CBI-model, and the topical and intradermal capsaicin models [2]. Heat conditioning had no effect on primary hyperalgesia or the SHAs in any of the models but produced minor changes in local skin temperature, area of flare, and skin blood flow [2].

When comparing the CBI-model (330 s at 45°C, 10.2 cm^2^) to the topical capsaicin and UVB model [3], the capsaicin and UVB models both increased primary hyperalgesia to heat and mechanical stimuli to a higher degree than the CBI-model. Topical capsaicin-induced larger SHAs and flare than developed in the CBI-model, while the UVB model produced no measurable area of flare or SHA [3].

## **The contact burn injury in comparison to other cutaneous heat pain models**

The present review only included studies applying the CBI-model. However, other cutaneous heat pain models exist, differing in methodology, skin sensitization, and pharmacodynamic profiles [4]. The following table provides an overview of these models, however, a detailed comparison, nevertheless, is beyond the scope of this review.

**Table. Comparison of Miscellaneous Cutaneous Heat Pain Models.**

|  | Contact burn injury | Heat/capsaicin | UVB |
| --- | --- | --- | --- |
| **Methodology** | Heating by a contact thermode | Heating by a contact thermode (300 s at 45°C), followed by topical capsaicin and heat rekindling | UVB irradiation |
| **Sensory changes** | Primary and secondary hyperalgesia; facilitation of temporal summation; duration of hyperalgesia depends on the intensity of the CBI | Primary and secondary hyperalgesia; facilitation of temporal summation; duration around 3-4 h, obtained by repeated rekindling [5,6]; hyperalgesia is dependent on size, duration, and intensity of the noxious stimulus [7] | Dose-dependent primary hyperalgesia to heat and mechanical stimulation with a peak at 24 h [3]; Secondary hyperalgesia is an inconsistent finding [3,8] |
| **Validated?** | Yes [9,10] | Yes [5] | Yes [11] |
| **Pharmacodynamic profile** | Moderately sensitive to ketamine; limited sensitivity to opioids, NSAIDs, glucocorticoids | Effect of opioids, gabapentin, and NMDA receptor antagonists; weak to no effect of NSAIDs, adenosine and lidocaine* | Very sensitive to NSAIDs; moderately sensitive to opioids; some effect of lidocaine; weak to no effect of benzodiazepines, gabapentin, paracetamol, botulinum toxin A, and tetrahydrocannabinol* |
| **Adverse events** | Pain and discomfort during CBI-induction; risk of blistering depending on heating paradigm | Pain and discomfort during heating and capsaicin [6]; minimal risk of blistering | No pain during UVB irradiation and no blistering or skin damage [11] |

*Based in part on the work by van Amerongen et al. [4], see reference for a more detailed description of outcomes; **CBI** = contact burn injury, **NMDA** = *N*-methyl-D-aspartate, **NSAID** = Non-steroidal Anti-inflammatory Drug, **UVB** = ultraviolet B.

1. Yucel A, Miyazawa A, Andersen OK, Arendt-Nielsen L. Comparison of hyperalgesia induced by capsaicin injection and controlled heat injury: Effect on temporal summation. Somatosens Mot Res. 2004;21(1):15-24. doi: 10.1080/0899022042000201263.

2. Yucel A, Miyazawa A, Andersen OK, Arendt-Nielsen L. The effect of heat conditioning of the primary area before and after induction of hyperalgesia by topical/intradermal capsaicin or by controlled heat injury. Somatosens Mot Res. 2001;18(4):295-302. doi: 10.1080/01421590120089677.

3. Bishop T, Ballard A, Holmes H, Young AR, McMahon SB. Ultraviolet-B induced inflammation of human skin: characterisation and comparison with traditional models of hyperalgesia. Eur J Pain. 2009;13(5):524-32. doi: 10.1016/j.ejpain.2008.06.006.

4. van Amerongen G, de Boer MW, Groeneveld GJ, Hay JL. A literature review on the pharmacological sensitivity of human evoked hyperalgesia pain models. Br J Clin Pharmacol. 2016:903-22. doi: 10.1111/bcp.13018.

5. Dirks J, Petersen KL, Dahl JB. The heat/capsaicin sensitization model: a methodologic study. J Pain. 2003;4(3):122-8. doi: 10.1054/jpai.2003.10.

6. Petersen KL, Rowbotham MC. A new human experimental pain model: The heat/capsaicin sensitization model. NeuroReport. 1999;10(7):1511-6. doi: 10.1097/00001756-199905140-00022.

7. Cavallone LF, Frey K, Montana MC, Joyal J, Regina KJ, Petersen KL, et al. Reproducibility of the heat/capsaicin skin sensitization model in healthy volunteers. J Pain Res. 2013;6:771-84. doi: 10.2147/jpr.S53437. PubMed Central PMCID: PMCPMC3827105.

8. Gustorff B, Anzenhofer S, Sycha T, Lehr S, Kress HG. The sunburn pain model: the stability of primary and secondary hyperalgesia over 10 hours in a crossover setting. Anesth Analg. 2004;98(1):173-7, table of contents. doi: 10.1213/01.ane.0000093224.77281.a5.

9. Norbury TA, MacGregor AJ, Urwin J, Spector TD, McMahon SB. Heritability of responses to painful stimuli in women: a classical twin study. Brain. 2007;130(Pt 11):3041-9. doi: 10.1093/brain/awm233.

10. Pedersen JL, Kehlet H. Hyperalgesia in a human model of acute inflammatory pain: A methodological study. Pain. 1998;74(2-3):139-51. doi: 10.1016/s0304-3959(97)00160-7.

11. Morch CD, Gazerani P, Nielsen TA, Arendt-Nielsen L. The UVB cutaneous inflammatory pain model: A reproducibility study in healthy volunteers. Int J Physiol Pathophysiol Pharmacol. 2013;5(4):203-15.
