# Supplementary material for: Methodology and applicability of the human contact burn injury model: A systematic review
Source: PLoS One. 2021 Jul 30;16(7):e0254790. doi: 10.1371/journal.pone.0254790 (PMC8323928; doi:10.1371/journal.pone.0254790)
Supplement: Attachment — Submitted filename: PONE-D-20-38717_Comments_Authors.pdf [file pone.0254790.s006.pdf]

PONE-D-20-38717 Reviewer Comments

Comments to Authors

General comments:

This manuscript provides a very thorough overview and summary of the contact burn injury model. The background section of the introduction is well written and succinct, however following sections providing additional background are lengthy and could be condensed and incorporated into the introduction, methods, and discussion. The methods are well written, however, substantially more information is needed on the quality assessment of articles. The results are rather repetitive, and the discussion is lengthy, and could be condensed. My main concern with the manuscript is the lack of removal of low-quality intervention studies. It is unclear to what end the author's main findings are derived from low quality studies compared to high quality. I would suggest incorporating a more critical assessment of the literature throughout the discussion.

Specific comments:

Line 40-44: The first sentence required 64 references, yet the second and, more informative sentence, remains unreferenced?

Sections "Classification of cutaneous burns", "Contact burn injury method", and "Evaluation of contact burn injury-induced changes" are all rather lengthy and feel disjointed between the introduction and the methods. Consider condensing these sections to incorporating them into the discussion, such that following the purpose of the review, the manuscript leads seamlessly into the methods.

With numerous acronyms, consider removing SHA. Numerous times throughout the manuscript, secondary hyperalgesia is mentioned but not short formed, while SHA is short formed. This feels rather odd when reading.

Line 208-210. 73 records assessed for eligibility, 12 excluded, 5 added, to total 64 does not add up. (73-12+5=66)

The authors do not discuss their quality assessment until the limitations section of the review. Why are these not further discussed in greater detail throughout the manuscript? The methods do not provide enough detail to interpret the quality outcomes (i.e. what does a score of 2 mean?). To that end, authors suggest over half of the intervention studies received a score of 0 on their chosen scale. What does a score of 0 represent? The authors have not provided information to interpret these values given in their tables. Additionally, this scale is more lenient than Cochrane's suggested tool. Why were so many studies with quality such low quality included in the review and the discussion? The justification that "most were hypothesis driven pragmatic studies" does not accommodate for both methodological quality or author bias. This needs to be better addressed in the results and discussion.

I'm curious as to why the authors did not perform any meta-analysis of findings? With the high number of included studies, it would have been beneficial for some of the more common outcomes to be included in separate meta analyses.

Line 785 – what about capsaicin as well? The heat capsaicin model is very well documented.

Table 6 – These seem to be rather vague recommendations for future studies. Specifically, how long should stimulus be applied? To what temperature? These details would be expected in a summary table outlining the updated recommended methodology, as alluded to in the abstract.
