# Supplementary material for: Methodology and applicability of the human contact burn injury model: A systematic review
Source: PLoS One. 2021 Jul 30;16(7):e0254790. doi: 10.1371/journal.pone.0254790 (PMC8323928; doi:10.1371/journal.pone.0254790)
Supplement: Attachment — Submitted filename: Response to Reviewers.docx [file pone.0254790.s007.docx]

**RESPONSE LETTER TO THE EDITOR AND THE REVIEWERS**

PONE-D-20-38717
Methodology and applicability of the human contact burn injury model: A systematic review

The following is a point-by-point response to the Editor’s comments (part **A**), and to the Reviewers’ queries (part **B**). In both parts, the authors’ replies are in blue font color. In part **B**, the Reviewers’ queries, in black font color, are numbered consecutively and chronologically, i.e., **Q1**, **Q2**, **Q3**, etc. The authors’ replies are correspondingly numbered, i.e., **R1**, **R2**, **R3**, etc.

Amendments to the manuscript text have been marked in red font color, and deletions from the manuscript text are marked with a strikethrough. Indicated line numbers (Page XX, Line YY-ZZ) refer to the text in the unmarked version of the revised manuscript without tracked changes.

**A. Comments to the Editor**

JOURNAL REQUIREMENTS:

**1**. The authors have ensured that the manuscript meets PLOS ONE style requirements, including those for file naming.

ADDITIONAL EDITOR COMMENTS:

Thank you for submitting your manuscript to PLOS ONE. The comments from the reviewers are attached. Overall, the manuscript has potential to serve as a valuable resource for researchers interested in the contact burn injury model of inducing primary and secondary hyperalgesia. My primary concern, however, is that the manuscript is burdened by redundancies, which makes it rather overwhelming to read and interpret. I would ask that the authors consider the comments of the reviewers and in turn attempt to streamline the results section accordingly, condensing where possible.

We have done our utmost to comply with the Reviewers’ comments, thus streamlining the manuscript wherever possible. The manuscript’s overall word count has been reduced with 21% compared with the original submission.

In addition, I would request that the authors confirm they searched for previously published systematic reviews/meta-analyses on the topic of contact burn injuries. This needs to be done to avoid duplicate efforts and multiple reviews written needlessly on the same topic. If there are none, please indicate as such in the manuscript. Finally, please consider using the AMSTAR tool to evaluate your systematic review and ensure it meets the highest methodological standard.

During our literature search, we have looked for systematic reviews on PubMed and PROSPERO on topics related to contact burn injuries. The single study we retrieved has already been mentioned in our initially submitted manuscript [1]. However, we now explicitly state that we performed this search in the manuscript and have added the following sentence:

- Page 9, Line 168-172: ‘Before commencing the literature search, the authors searched for similar reviews in the field of contact burn injuries on PubMed and PROSPERO. We found one published review from 2016 regarding the pharmacological sensitivity of the CBI model [1]. This study did not focus on methodological aspects of the CBI model in detail.’

As suggested, the authors applied the AMSTAR tool in order to optimize the methodological quality of the review.

During the revision of the manuscript, the authors detected an unfortunate error in the Abstract which is now corrected.

- Page 2, Line 23: “Sixty-three studies were included” has been changed to ‘Sixty-four studies were included.’

**B. Comments to the Reviewers**

The authors acknowledge with gratitude the Reviewers’ effort in preparing the evaluation of the manuscript. The authors consider the suggestions and queries for having improved the manuscript significantly. The authors are very grateful to the Reviewers.

**REVIEWER #1**:

This manuscript provides a very thorough overview and summary of the contact burn injury model. The background section of the introduction is well written and succinct, however following sections providing additional background are lengthy and could be condensed and incorporated into the introduction, methods, and discussion. The methods are well written, however, substantially more information is needed on the quality assessment of articles. The results are rather repetitive, and the discussion is lengthy, and could be condensed. My main concern with the manuscript is the lack of removal of low-quality intervention studies. It is unclear to what end the author’s main findings are derived from low quality studies compared to high quality. I would suggest incorporating a more critical assessment of the literature throughout the discussion.

The authors thank the Reviewer for these positive comments. We have rewritten the Introduction and, as suggested, incorporated the additional background information in other sections. Further, we have provided additional information on quality assessment in the Material and method section. The Result and Discussion sections have been abridged substantially, i.e., 17% and 22%, respectively. The authors have put a great deal of effort into shortening the sections without losing the sense of coherence.

To improve the reading experience the authors have moved some of the information to supporting information. This includes:

- Statistical definitions (former Table 4) to ‘S1 Table. Statistical definitions’

- Results from miscellaneous studies to ‘S2 File. Results from the “miscellaneous” studies’

- Results regarding comparison to other cutaneous heat pain models to ‘S3 File. The contact burn injury model in comparison to other cutaneous heat pain models

COMMENTS:

**Q1:** Line 40-44: The first sentence required 64 references, yet the second and, more informative sentence, remains unreferenced?

**R1:** The in-text references #1-64, comprising all the studies of the present review, were actually a technical feature facilitating a chronological order of the study material from the beginning. In the second sentence of the paragraph, three references have been added [1-3] (Page 3, Line 42-44).

**Q2:** Sections “Classification of cutaneous burns”, “Contact burn injury method”, and “Evaluation of contact burn injury-induced changes” are all rather lengthy and feel disjointed between the introduction and the methods. Consider condensing these sections to incorporating them into the discussion, such that following the purpose of the review, the manuscript leads seamlessly into the methods.

**R2:** We agree that the subsections in the Introduction section could be incorporated elsewhere. We now have a single conjoined Introduction, where the following paragraph has been added:

- Page 3-4, Line 51-68: ‘The CBI-model involves tonic heating of the skin by a contact thermode inducing a first-degree burn injury. Critical factors that govern cutaneous burn injury depth are exposure temperature and exposure time, a relationship first proposed by Moritz and Henriques in 1947 [4]. In their hallmark experimental study, porcine and human skin was exposed to a contact thermode with varying exposure temperatures and times in order to establish a time-temperature graph. The generated graph indicated the lowest exposure time at a given temperature, causing microscopically verified epidermal cell death (Fig 2). Within the range of 44°C to 51°C, an increase in heating of 1°C meant that the exposure time required for the development of epidermal cell death was reduced by 50%. At 44°C, an exposure time of 6 h was required to induce epidermal cell death; thus, the authors concluded that, below this temperature, cellular reparative processes were able to prevent burn injury damage [4]. Further studies have confirmed that heating the skin at 45°C for 300 s only induces transient hyperalgesia [5-7].

The application of the CBI-model generally involves the CBI as a conditioning stimulus with pain intensity ratings during the induction followed by Quantitative Sensory Testing, i.e., a standardized activation of the sensory system by application of graded chemical, electrical, mechanical, or thermal stimuli, with an assessment of the evoked psychophysical responses [8].’

The rest of the information in the mentioned sections has been condensed and added in the Discussion section with the following edits:

- Page 44, Line 528-533: ‘As previously reported, Moritz and Henriques [4] described first-degree burn injuries ranging from transient hyperemia to ~~severe and~~ prolonged erythema with the formation of small vesicles [4]. Erythema reflects vascular changes in the burn area, and the degree of redness is measured by skin reflectance spectrophotometry [9]. The duration of erythema has varied substantially across CBI-studies. Other indices of inflammation, i.e., increased dermal blood flow, a rise in skin temperature, and ~~including development of~~ flare ~~and increase in skin temperature and dermal blood flow~~, are only short-lasting phenomena post-CBI [10-14].’

- Page 44, Line 538-544: ‘Assessing the central anti-hyperalgesic effect, by evaluating changes in SHA, is an essential outcome of many analgesic studies. ~~Assessing SHAs is an essential part of phase 1 and 2 studies, evaluating the central effects of analgesics.~~ The SHA is delineated via punctate stimulation with a nylon filament or a weighted-pin instrument. The CBI induces rather consistent secondary hyperalgesia, with a duration of 24 h. Interestingly, two studies have indicated that ~~reported an inability to assess SHA in a proportion of subjects following the CBI. A total of 12%~~ 12/100 (420 s at 47°C, 12.5 cm^2^) [15], and 10/64 (300 s at 46°C, 12.5 cm^2^) [16] of the subjects do not develop measurable SHAs. Paradoxically, QST assessments by themselves may induce secondary hyperalgesia [17,18].’

**Q3**: With numerous acronyms, consider removing SHA. Numerous times throughout the manuscript, secondary hyperalgesia is mentioned but not short formed, while SHA is short formed. This feels rather odd when reading.

**R3:** The authors agree that it is inconsequential to switch between ‘secondary hyperalgesia area’ and ‘SHA’. However, since ‘SHA’ is mentioned numerous times (41 in the main text of the revised manuscript), we have changed ‘secondary hyperalgesia area’ to ‘SHA’ in all relevant places. There is, however, a distinction between ‘secondary hyperalgesia’ and ‘secondary hyperalgesia area’, and in order to improve readability, the authors have kept ‘secondary hyperalgesia’ unabbreviated.

**Q4:** Line 208-210. 73 records assessed for eligibility, 12 excluded, 5 added, to total 64 does not add up. (73-12+5=66)

**R4:** The authors thank the Reviewer for pointing out this error. We excluded 14 studies and not 12. This has been changed now.

**Q5:** The authors do not discuss their quality assessment until the limitations section of the review. Why are these not further discussed in greater detail throughout the manuscript?

**R5:** Thank you for raising a relevant objection. Quality assessment using the Oxford Quality Scoring System can only be performed on RCTs. Therefore, quality assessment scores have only been made for the intervention studies and are thus only relevant when discussing these studies. However, we agree that more information could be provided about the quality scores. Consequently, the authors have put more effort into discussing quality scoring throughout the manuscript and have added additional information about quality scoring in the limitations sub-section:

- Page 48, Line 613-620: ‘The quality score depends on the information available in the reports, and journal requirements for trial reporting have not always been as comprehensive as today [19]. Furthermore, the scoring systems used in systematic reviews and meta-analyses, albeit important, only characterize certain aspects of the research design [20,21]. The quality scorings, therefore, may be difficult to interpret in a review, including studies of different time epochs. Thus, the individual studies may have been performed well regarding the somatosensory testing methodology, the main objective of the present review, nevertheless, receiving a low-quality rating. Valuable methodological information could have been neglected by excluding studies based on quality scores.’

**Q6:** The methods do not provide enough detail to interpret the quality outcomes (i.e. what does a score of 2 mean?). To that end, authors suggest over half of the intervention studies received a score of 0 on their chosen scale. What does a score of 0 represent? The authors have not provided information to interpret these values given in their tables. Additionally, this scale is more lenient than Cochrane’s suggested tool. Why were so many studies with quality such low quality included in the review and the discussion? The justification that “most were hypothesis driven pragmatic studies” does not accommodate for both methodological quality or author bias. This needs to be better addressed in the results and discussion.

**R6:** The authors thank the Reviewer for these suggestions. We have added additional information in the method section about the interpretation of the quality scores (please, also cf. **R5** above):

- Page 8, Line 151-155: ‘Methodological quality was assessed using the Oxford Quality Scoring System [22] for all intervention studies. This system evaluates information about randomization, blinding, and withdrawals, and drop-outs. Each study is scored on an ordinal scale of 0-5, with 0-2 representing a low-quality score and 3-5 representing a high-quality score.’

During the revision of the manuscript, the authors became aware of an erroneous scoring procedure of the papers. The Oxford Quality Scoring System evaluates information about randomization, blinding, and subject dropouts. Studies get subtraction of a point if the randomization process and/or the blinding process was described and was inadequate. However, in our initial scoring of the studies, we subtracted a point if the information was missing. This resulted in a lot of studies receiving a score of 0 even though they were described as randomized, double-blind studies. We believe this may have given an inaccurate picture of the quality of the studies. The quality scores have, thus, been updated.

As mentioned in **R5**, we have updated the discussion regarding the quality scores and the inclusion of low-quality studies. We believe that valuable information on methodological aspects of the somatosensory testing would potentially have been lost by excluding intervention studies based on quality scores.

**Q7:** I’m curious as to why the authors did not perform any meta-analysis of findings? With the high number of included studies, it would have been beneficial for some of the more common outcomes to be included in separate meta analyses.

**R7:** In order to perform a meta-analysis, the authors would have had to look at a number of studies employing the same intervention and using identical somatosensory testing methodology. However, given the heterogeneity of the CBI methodologies applied, we did not find it meaningful to perform a meta-analysis.

We have updated the discussion about performing a meta-analysis:

- Page 48, Line 621-624: ‘Another essential point is that the included studies demonstrated a high heterogeneity in regard to the methodology of the CBI-model and associated outcome assessments. Consequently, a statistical meta-analysis of the included results was not deemed feasible and was not within the scope of this review.’

**Q8:** Line 785 – what about capsaicin as well? The heat capsaicin model is very well documented.

**R8:** The Reviewer can rightfully claim that the authors could have mentioned the well documented heat-capsaicin model here. We have changed the sentence now in the following way:

- Page 49, Line 648-650: ‘However, the inflammatory response could instead be augmented synergistically by topical administration of pro-inflammatory agents such as mustard oil [23], sodium lauryl sulfate [24], or the well-investigated capsaicin [5], enhancing the primary and secondary hyperalgesia components.’

**Q9:** Table 6 – These seem to be rather vague recommendations for future studies. Specifically, how long should stimulus be applied? To what temperature? These details would be expected in a summary table outlining the updated recommended methodology, as alluded to in the abstract.

**R9:** We agree with the Reviewer. The information in Table 4 has now been condensed, providing the most relevant methodological information available (Page 50-51):

**Table 4. Suggestions to consider when designing a CBI-model trial.**

**CBI-methodology**

1) Equipment calibration
2) Heating paradigm: 420 s at 47.0°C; ≥ 9 cm^2^
3) Induction site: lower arm or leg

**Outcome assessments**

4) Pain during the CBI
5) Primary hyperalgesia
   - Mechanical/thermal thresholds
   - Mechanical/thermal suprathreshold stimuli
   - Temporal summation
6) Secondary hyperalgesia
   - Mechanical delineation of SHAs
   - Temporal summation
7) Inflammatory variables?
   - Erythema
   - Edema

**Pharmacodynamic considerations**

8) Measurement of subjects’ reaction times (‘method of limits’)
9) Use suitable dose-response design

**Statistical considerations**

10) Consider obtaining test-retest data

Authors’ considerations, based on the review, when designing a contact burn injury (CBI)-model trial. **1**) Calibration of the CBI-equipment is mandatory. **2**) The most commonly used heating paradigm (the most commonly used heating area was 12.5 cm^2^). **3**) The induction sites are the most frequently used. **4**) CBI-induced pain can be used both as conditioning- and test-stimulus. **5**) In the CBI-area assessment of thresholds, pain perception to suprathreshold stimuli or temporal summation can be used. **6**) The secondary hyperalgesia area (SHA) is delineated by mechanical stimuli [25], and perception to temporal summation can be evaluated as central sensitization measures. **7**) CBI-induced inflammatory variables to be considered are erythema and edema [26]. **8**) Analgesic trials, using the ‘method of limits’ in threshold assessments, may require assessment of the subjects’ reaction times in order to avoid ‘pseudo-analgesia’ [27]. **9**) Dose-response studies require a design with multiple doses [28]. **10**) In order to validate the study methodology, a test-retest set-up should be considered [29].

**REVIEWER #2**

 This systematic review by Springborg and colleagues aimed to synthesize data from studies using human burn injury models. This is an important work and, for the most part, provides a nice overview of many human model parameters as applied in the literature. It is very comprehensive, which introduces some difficulties in reporting clear, concise and synthesized results due to obvious variation between studies.

The authors thank the Reviewer for the positive comments. We have made an effort to abridge and streamline the manuscript further.

COMMENTS:
**Q1:** Page 12, line 125: "QST…….by application of graded chemical, electrical,…." What chemical and electrical stimuli are the authors referring to? To the reviewer`s knowledge, classical QST protocols do not employ such stimuli.

**R1:** The authors acknowledge that ‘classic’ QST-assessments mainly involve thermal or mechanical stimulations. However, several studies, including some studies from the current review, employ electrical stimuli [30-32] or chemical stimuli [14,30].

**Q2:** I would like to commend the authors on their consistent use of terminology, as they are certainly well aware of the ongoing controversy regarding 'central sensitization' being used as a broad term in human studies in contrast to the very well-defined neuronal construct in basic animal studies. I am glad to see that the psychophysical terminology was used and distinctions between primary and secondary hyperalgesia were made appropriately. This was especially important for the interventional trials as some drugs will affect the primary input and the 2HA, respectively.

However, with respect to the "timing of intervention" it would be interesting to know if the studies aimed at investigating whether the drugs affected the induction of hyperalgesia or lead to a reversal of an already established state of hyperalgesia? In my personal opinion, only the former would constitute a true "anti-hyperalgesic effect". The latter could just be called "analgesic effect" considering CBI as a human surrogate pain model.

**R2:** Thank you very much for the relevant question. The authors agree that the timing of the intervention is an important factor potentially affecting the attenuation of hyperalgesia. The timing of interventions has indeed already been indicated for the individual studies in Table 3, as well as in the ‘Study outcomes – Intervention studies’ sub-section in the Results. We have, however, made some slight changes:

- Page 30, Line 369-370: ‘The effects of ketamine on SHAs were observed regardless of whether it was administered preemptively [33-35] or post-CBI [36].’

**Q3:** In table 6 the use of "assessments of central mechanisms" is recommended. These tests are merely listed in the outcomes section in the introduction.
Please explain the methods/paradigms in a bit more detail, especially with regard to what mechanisms are potentially investigated with the respective paradigms.

**R3:** The assessments of central mechanisms refer predominantly to an evaluation of the development of secondary hyperalgesia and to a change in the response to temporal summation. Describing the specific QST outcomes is not within the scope of this review, and we refer to additional literature on this subject. However, the results regarding secondary hyperalgesia and temporal summation have been described throughout the manuscript. As mentioned in **R9** (Reviewer #1) we have updated Table 6 (now Table 4) and condensed the information to only include the most important methodological information.

REFERENCES

1.	van Amerongen G, de Boer MW, Groeneveld GJ, Hay JL. A literature review on the pharmacological sensitivity of human evoked hyperalgesia pain models. Br J Clin Pharmacol. 2016:903-22. doi: 10.1111/bcp.13018.

2.	Siebenga PS, van Amerongen G, Okkerse P, Denney WS, Dua P, Butt RP, et al. Reproducibility of a battery of human evoked pain models to detect pharmacological effects of analgesic drugs. Eur J Pain. 2019;23(6):1129-40. doi: 10.1002/ejp.1379. PubMed Central PMCID: PMCPMC6618124.

3.	Loudon P, Siebenga P, Gorman D, Gore K, Dua P, van Amerongen G, et al. Demonstration of an anti-hyperalgesic effect of a novel pan-Trk inhibitor PF-06273340 in a battery of human evoked pain models. Br J Clin Pharmacol. 2018;84(2):301-9. doi: 10.1111/bcp.13448. PubMed Central PMCID: PMCPMC5777446.

4.	Moritz AR, Henriques FC. Studies of Thermal Injury: II. The Relative Importance of Time and Surface Temperature in the Causation of Cutaneous Burns. Am J Pathol. 1947;23(5):695-720. PubMed Central PMCID: PMCPMC1934304.

5.	Petersen KL, Rowbotham MC. A new human experimental pain model: The heat/capsaicin sensitization model. NeuroReport. 1999;10(7):1511-6. doi: 10.1097/00001756-199905140-00022.

6.	Dirks J, Petersen KL, Dahl JB. The heat/capsaicin sensitization model: a methodologic study. J Pain. 2003;4(3):122-8. doi: 10.1054/jpai.2003.10.

7.	Brennum J, Dahl JB, Moiniche S, Arendt-Nielsen L. Quantitative sensory examination of epidural anaesthesia and analgesia in man: Effects of pre- and post-traumatic morphine on hyperalgesia. Pain. 1994;59(2):261-71. doi: 10.1016/0304-3959(94)90079-5.

8.	Rolke R, Magerl W, Campbell KA, Schalber C, Caspari S, Birklein F, et al. Quantitative sensory testing: A comprehensive protocol for clinical trials. Eur J Pain. 2006;10(1):77-88. doi: 10.1016/j.ejpain.2005.02.003.

9.	Bjerring P, Andersen PH. Skin reflectance spectrophotometry. Photodermatol. 1987;4(3):167-71.

10.	Schmelz M, Michael K, Weidner C, Schmidt R, Torebjork HE, Handwerker HO. Which nerve fibers mediate the axon reflex flare in human skin? Neuroreport. 2000;11(3):645-8. doi: 10.1097/00001756-200002280-00041.

11.	Janig W, Lisney SJ. Small diameter myelinated afferents produce vasodilatation but not plasma extravasation in rat skin. J Physiol. 1989;415:477-86. doi: 10.1113/jphysiol.1989.sp017732. PubMed Central PMCID: PMCPMC1189187.

12.	Lewis T. Nocifensor System of Nerves. Br Med J. 1937;1(3973):431-5. PubMed Central PMCID: PMCPMC2088263.

13.	Schulte H, Sollevi A, Segerdahl M. The distribution of hyperaemia induced by skin burn injury is not correlated with the development of secondary punctate hyperalgesia. J Pain. 2004;5(4):212-7. doi: 10.1016/j.jpain.2004.03.002.

14.	Yucel A, Miyazawa A, Andersen OK, Arendt-Nielsen L. The effect of heat conditioning of the primary area before and after induction of hyperalgesia by topical/intradermal capsaicin or by controlled heat injury. Somatosens Mot Res. 2001;18(4):295-302. doi: 10.1080/01421590120089677.

15.	Ravn P, Frederiksen R, Skovsen AP, Christrup LL, Werner MU. Prediction of pain sensitivity in healthy volunteers. J Pain Res. 2012;5:313-26. doi: 10.2147/JPR.S33925.

16.	Matre D, Casey KL, Knardahl S. Placebo-induced changes in spinal cord pain processing. J Neurosci. 2006;26(2):559-63. doi: 10.1523/jneurosci.4218-05.2006.

17.	Pedersen JL, Kehlet H. Hyperalgesia in a human model of acute inflammatory pain: A methodological study. Pain. 1998;74(2-3):139-51. doi: 10.1016/s0304-3959(97)00160-7.

18.	Cervero F, Gilbert R, Hammond RG, Tanner J. Development of secondary hyperalgesia following non-painful thermal stimulation of the skin: a psychophysical study in man. Pain. 1993;54(2):181-9. doi: 10.1016/0304-3959(93)90207-6.

19.	Jadad AR, Moore RA, Carroll D, Jenkinson C, Reynolds DJ, Gavaghan DJ, et al. Assessing the quality of reports of randomized clinical trials: is blinding necessary? Control Clin Trials. 1996;17(1):1-12.

20.	Ioannidis J. Next-generation systematic reviews: prospective meta-analysis, individual-level data, networks and umbrella reviews. Br J Sports Med. 2017;51(20):1456-8. doi: 10.1136/bjsports-2017-097621.

21.	Kehlet H, Joshi GP. Systematic Reviews and Meta-Analyses of Randomized Controlled Trials on Perioperative Outcomes: An Urgent Need for Critical Reappraisal. Anesth Analg. 2015;121(4):1104-7. doi: 10.1213/ANE.0000000000000687 [doi];00000539-201510000-00033 [pii].

22.	Jadad AR, Moore RA, Carroll D, Jenkinson C, Reynolds DJ, Gavaghan DJ, et al. Assessing the quality of reports of randomized clinical trials: is blinding necessary? Control Clin Trials. 1996;17(1):1-12. doi: 10.1016/0197-2456(95)00134-4.

23.	Koltzenburg M, Lundberg LE, Torebjork HE. Dynamic and static components of mechanical hyperalgesia in human hairy skin. Pain. 1992;51(2):207-19. doi: 10.1016/0304-3959(92)90262-a.

24.	Petersen LJ, Lyngholm AM, Arendt-Nielsen L. A novel model of inflammatory pain in human skin involving topical application of sodium lauryl sulfate. Inflamm Res. 2010;59(9):775-81. doi: 10.1007/s00011-010-0189-1.

25.	Ringsted TK, Enghuus C, Petersen MA, Werner MU. Demarcation of secondary hyperalgesia zones: Punctate stimulation pressure matters. J Neurosci Methods. 2015;256:74-81. doi: S0165-0270(15)00309-X [pii];10.1016/j.jneumeth.2015.08.018 [doi].

26.	Andersen LP, Gogenur I, Fenger AQ, Petersen MC, Rosenberg J, Werner MU. Analgesic and anti-hyperalgesic effects of melatonin in a human inflammatory pain model: a randomized, double-blind, placebo-controlled, three-arm crossover study. Pain. 2015. doi: 10.1097/j.pain.0000000000000284 [doi].

27.	Ravn P, Secher EL, Skram U, Therkildsen T, Christrup LL, Werner MU. Morphine- and buprenorphine-induced analgesia and antihyperalgesia in a human inflammatory pain model: a double-blind, randomized, placebo-controlled, five-arm crossover study. J Pain Res. 2013;6:23-38. doi: 10.2147/JPR.S36827 [doi];jpr-6-023 [pii].

28.	Shafer SL. The importance of dose-response in study design. Anesth Analg. 1999;89(3):805.

29.	Werner MU, Petersen MA, Bischoff JM. Test-retest studies in quantitative sensory testing: a critical review. Acta Anaesthesiol Scand. 2013;57(8):957-63. doi: 10.1111/aas.12150 [doi].

30.	Yucel A, Miyazawa A, Andersen OK, Arendt-Nielsen L. Comparison of hyperalgesia induced by capsaicin injection and controlled heat injury: Effect on temporal summation. Somatosens Mot Res. 2004;21(1):15-24. doi: 10.1080/0899022042000201263.

31.	Pedersen JL, Andersen OK, Arendt-Nielsen L, Kehlet H. Hyperalgesia and temporal summation of pain after heat injury in man. Pain. 1998;74(2-3):189-97. doi: 10.1016/s0304-3959(97)00162-0.

32.	Drummond PD. Depletion of noradrenaline inhibits electrically-evoked pain in the skin of the human forearm. Eur J Pain. 2008;12(2):196-202. doi: 10.1016/j.ejpain.2007.05.001.

33.	Ilkjaer S, Petersen KL, Brennum J, Wernberg M, Dahl JB. Effect of systemic N-methyl-D-aspartate receptor antagonist (ketamine) on primary and secondary hyperalgesia in humans. Br J Anaesth. 1996;76(6):829-34. doi: 10.1093/bja/76.6.829.

34.	Warncke T, Stubhaug A, Jorum E. Preinjury treatment with morphine or ketamine inhibits the development of experimentally induced secondary hyperalgesia in man. Pain. 2000;86(3):293-303. doi: 10.1016/s0304-3959(00)00260-8.

35.	Warncke T, Jorum E, Stubhaug A. Local treatment with the N-methyl-D-aspartate receptor antagonist ketamine, inhibit development of secondary hyperalgesia in man by a peripheral action. Neurosci Lett. 1997;227(1):1-4. doi: 10.1016/s0304-3940(97)00263-2.

36.	Warncke T, Stubhaug A, Jorum E. Ketamine, an NMDA receptor antagonist, suppresses spatial and temporal properties of burn-induced secondary hyperalgesia in man: A double-blind, cross-over comparison with morphine and placebo. Pain. 1997;72(1-2):99-106. doi: 10.1016/s0304-3959(97)00006-7.
