# Supplementary material for: Methodology and applicability of the human contact burn injury model: A systematic review
Source: PLoS One. 2021 Jul 30;16(7):e0254790. doi: 10.1371/journal.pone.0254790 (PMC8323928; doi:10.1371/journal.pone.0254790)
Supplement: Attachment — Submitted filename: Response to Reviewers.docx [file pone.0254790.s008.docx]

**RESPONSE LETTER TO THE EDITOR AND THE REVIEWERS**

PONE-D-20-38717R1
Methodology and applicability of the human contact burn injury model: A systematic review

The following is a point-by-point response to the Editor’s comments (part **A**) and to the Reviewers’ queries (part **B**). In both parts, the authors’ replies are in blue font color. In part **B**, the Reviewers’ queries are numbered consecutively and chronologically, i.e., **Q1**, **Q2**, **Q3**, etc. The authors’ replies are correspondingly numbered, i.e., **R1**, **R2**, **R3**, etc.

Amendments to the manuscript text have been marked in red font color, and deletions from the manuscript text are marked with a strikethrough. Indicated line numbers (Page XX, Line YY-ZZ) refer to the text in the unmarked version of the revised manuscript.

**A. Comments to the Editor**

JOURNAL REQUIREMENTS:

Please review your reference list to ensure that it is complete and correct. If you have cited papers that have been retracted, please include the rationale for doing so in the manuscript text, or remove these references and replace them with relevant current references. Any changes to the reference list should be mentioned in the rebuttal letter that accompanies your revised manuscript. If you need to cite a retracted article, indicate the article’s retracted status in the References list and also include a citation and full reference for the retraction notice.

The authors have reviewed the reference list again and have ensured that it is correct without any retracted papers.

**B. Comments to the Reviewers**

**REVIEWER #1**:

The authors have substantially improved the manuscript through the revision process. Many redundancies have been removed, improving the readability of the manuscript. The majority of my comments have been adequately addressed. I have a few minor comments for the authors consideration. The first should be very easy to include, and the remaining two may be worth consideration (or perhaps authors already considered this and sought not to include as it did not improve the interpretation).

The authors thank the reviewer for the kind comments and the help in improving the manuscript.

**Q1:** PRISMA Diagram and checklist. These are the old versions of the PRISMA diagram and checklist. These were both updated in 2020. Consider updated to keep in line with PRISMA guidelines.

**R1:** The authors acknowledge the reviewer for pointing this out. The updated versions of the PRISMA diagram and checklist from 2020 have been attached to the submission.

**Q2:** I previously suggested authors further discuss their quality assessment. Following revisions, the authors have pointed out that only intervention studies were assessed for quality. Are there no available tools to assess article quality for non-intervention studies? To that end, while the quality of intervention studies has been assessed, do the authors believe there would be any value in also using a tool to evaluate the quality of the data/findings from each study (using the GRADE tool perhaps)?

**R2:** There are several tools for assessing the quality of non-randomized studies. However, the authors have not been able to find a single tool that would be able to assess the quality of all the non-intervention studies in an intelligible fashion due to the considerable heterogeneity among these studies. Thus, the following sentence has been added to the Discussion:

- Page 48, Line 627-633: ‘Attempts were made at performing a quality scoring of the non-interventional studies. Incorporating study quality could provide clarity in studies presenting with contrasting results. Two potential scoring systems were considered: the GRADE tool [1,2] and the “checklist for the assessment of the methodological quality” [3]. However, due to considerable heterogeneity across the studies regarding methodology, outcome variables, and statistical processing, the authors decided to abandon the attempt to make a relevant quality scoring.’

**Q3:** Throughout the results and discussion, there is clearly much heterogeneity in study findings. The general sense from the review is there is still “much work to do” in terms of using this model. An example of this would be the perplexing findings of the non-responsiveness of the model to anti-inflammatory drugs, despite being an inflammatory model. One avenue to address these concerns may be in weighing the quality of findings (in terms of Oxford and GRADE tools) to reach some more definitive conclusions. As it stands now, the quality assessment for the most part appears to be for the reader’s information, rather than directly used to help guide the synthesis of findings. It would be interesting to see if incorporating study quality alongside such contrasting findings could provide some clarity in such circumstances.

**R3:** The authors agree that it might be interesting to review the different studies in light of their quality scores. However, there are still relatively few studies evaluating each drug group, and these studies use a very different methodology in terms of administration routes, outcome measures, etc. When reviewing each drug group, the general conclusion does not seem to change when only the high-quality studies are included (Oxford quality score 3-5). Thus, the authors have added the following sentence to the Discussion:

- Page 46-47, Page 590-597: ‘Interestingly, the studies evaluating anti-inflammatory drugs, i.e., NSAIDs and glucocorticoids, have mostly been negative but have received varying quality scores, raising the question of whether the results are correlated with the quality of the study. However, of the six studies evaluating NSAIDs, three different drugs and four different routes of administration were used along with several different outcome measures, obstructing a valid comparison between the studies. Further, among the three high-quality studies, only one study could be considered yielding a positive outcome [4], thus not changing the general conclusion of this review.’

**REVIEWER #2**: (No Response)

REFERENCES

1.	Atkins D, Best D, Briss PA, Eccles M, Falck-Ytter Y, Flottorp S, et al. Grading quality of evidence and strength of recommendations. BMJ. 2004;328(7454):1490. doi: 10.1136/bmj.328.7454.1490 [doi];328/7454/1490 [pii].

2.	Guyatt GH, Oxman AD, Vist GE, Kunz R, Falck-Ytter Y, Alonso-Coello P, et al. GRADE: an emerging consensus on rating quality of evidence and strength of recommendations. BMJ. 2008;336(7650):924-6. doi: 336/7650/924 [pii];10.1136/bmj.39489.470347.AD [doi].

3.	Downs SH, Black N. The feasibility of creating a checklist for the assessment of the methodological quality both of randomised and non-randomised studies of health care interventions. J Epidemiol Community Health. 1998;52(6):377-84. doi: 10.1136/jech.52.6.377. PubMed Central PMCID: PMCPMC1756728.

4.	Stubhaug A, Romundstad L, Kaasa T, Breivik H. Methylprednisolone and ketorolac rapidly reduce hyperalgesia around a skin burn injury and increase pressure pain thresholds. Acta Anaesthesiol Scand. 2007;51(9):1138-46. doi: 10.1111/j.1399-6576.2007.01415.x.
